# Exercise-induced attenuation of the lateral habenula: a multi-axial quantitative synthesis of circuit-level resilience in depression

**DOI:** 10.3389/fpsyt.2026.1887822

**Published:** 2026-08-04

**Authors:** Camila Ferreira-Vorkapic, Diego Moura Ferreira, Renato Ramos Coelho, Monika Anna Wiech, Estélio Henrique Martin Dantas

**Affiliations:** 1 Laboratory of Biosciences of Human Kinetics (LABIMH), PPGEnfBio\Federal University of the State of Rio de Janeiro (UNIRIO), Rio de Janeiro, Brazil; 2 Graduate Program in Nursing and Biosciences, Federal University of the State of Rio de Janeiro - UNIRIO, Rio de Janeiro, Brazil; 3 Unit Medical School, Tiradentes University - UNIT, Aracaju, Brazil; 4 Graduate Program in Nutritional Sciences (PPGCNUT), Federal University of Sergipe (UFS), Aracaju, Brazil; 5 Municipal Health Department of Contagem, Nomus Consulting, Brazilian Academic Consortium for Integrative Health (CABSIN), Laboratory of Biosciences of Human Kinetics (LABIMH), Federal University of the State of Rio de Janeiro (UNIRIO), Contagem, Brazil; 6 Gdansk University of Physical Education and Sport, Faculty of Physical Culture, Department of Sport, Gdansk, Poland; 7 Graduate Program in Biosciences and Healh - PBS, Tiradentes University - UNIT, Aracaju, Brazil

**Keywords:** anhedonia, depression, exercise, kynurenine, lateral habenula, NMHA model, RMTg

## Abstract

**Background:**

Depression is characterized by pathological lateral habenula (LHb) hyperactivity within the anti-reward circuitry. However, a unified framework explaining how exercise modulates this specific circuit remains to be established.Aims: To examine whether neurobiological evidence supports the Network-Mediated Habenular Attenuation (NMHA) Model, which proposes exercise as a systemic regulator of LHb pathology.

**Methods:**

A systematic synthesis was conducted following PRISMA guidelines and registered in PROSPERO. After a comprehensive search, 12 eligible studies met eligibility criteria. A quantitative synthesis was performed using a random-effects model, with Hedge’s *g* used to estimate the magnitude of neuroplastic changes across the NMHA axes.

**Results:**

Evidence suggests that exercise modulates LHb bursting via four convergent axes: (1) muscle-brain (peripheral-metabolic); (2) sensory-motor (monoaminergic); (3) cognitive-executive (top-down); and (4) hippocampal neurogenic (contextual). Quantitative synthesis revealed a robust global effect size (Hedges’*g* = 0.94; 95% CI [0.59, 1.29]). Heterogeneity was low (I^2^ = 14.90%). Some axes exhibited large effect sizes, with the hippocampal-contextual axis showing the highest potency (Hedges’*g* [1.95, 2.63]). These magnitudes provide a compensatory counter-signal to the pathological LHb burst firing observed in depressive states (Hedges’*g* ≈ 1.00), providing mathematical support for the NMHA model.

**Discussion:**

Exercise potentially acts as a circuit-breaker for anhedonia by modulating the anti-reward architecture. The observed variability in effect sizes (95% Prediction Interval: 0.21, 1.67) indicates that future studies may report effect sizes ranging from small to very large, reflecting the influence of different exercise protocols and depression models on circuit attenuation.

**Conclusion:**

The NMHA model positions exercise as a targeted neuromodulatory strategy. Future research should leverage high-field neuroimaging to translate these preclinical circuit-level findings into human clinical populations.

## Highlights

- Exercise acts as a systemic regulator of lateral habenula hyperactivity.
- Plasticity within the LHb-VTA circuit releases the inhibitory brake on dopamine.
- A random-effects quantitative synthesis confirms a robust global effect size (Hedges’g = 0.94) for LHb attenuation
- The hippocampal-contextual axis exhibits the highest statistical potency in modulating the anti-reward center (Hedges’g = 2.63).
- The NMHA Model provides a mechanistic framework for exercise as a targeted intervention for depression.

## Introduction

Depression is increasingly conceptualized not merely as a localized chemical imbalance, but as a disorder involving widespread dysregulation of circuit-level homeostasis. While traditional monoamine theories historically prioritized simple deficits in dopamine and serotonin availability, contemporary neuroimaging and electrophysiological evidence has identified the lateral habenula (LHb) as a central node within the anti-reward hub that drives the core symptoms of anhedonia and behavioral helplessness (1). In a healthy physiological state, the LHb provides the necessary feedback to calibrate reward-seeking behavior; however, in the depressed brain, this structure adopts a pathologically hyperactive, high-frequency burst firing pattern (2, 3). This aberrant electrical pattern exerts strong inhibitory control over the midbrain, specifically by recruiting the rostromedial tegmental nucleus (RMTg), the brain’s primary GABAergic “brake”, to suppress dopamine release in the ventral tegmental area (VTA) and serotonin within the raphe nuclei (4). These findings suggest that effective antidepressant interventions may require modulation of upstream anti-reward circuitry in addition to stimulation of reward-related regions.

### Neurobiological architecture of the lateral habenula: from homeostasis to pathology

To systematically evaluate how physical training interacts with the epithalamus, the baseline neurobiological properties, cellular diversity, and operational paradigms of the LHb must be explicitly characterized. At the cellular level, the LHb architectural landscape is predominantly comprised of glutamatergic projection neurons expressing vesicular glutamate transporter 2 (vGluT2). These neurons receive dense excitatory inputs from the limbic system, the entopeduncular nucleus/ventral pallidum, and the medial prefrontal cortex (mPFC), and subsequently funnel these signals down to primary monoaminergic centers via the canonical LHb-RMTg-VTA pathway (4, 5).

Under healthy physiological conditions, these vGluT2 projection neurons maintain a slow, irregular tonic firing pattern (~2–10 Hz). This baseline electrical activity serves a critical computational role, encoding negative prediction errors to systematically calibrate behavioral choices and reinforce active coping (1). In the depressive henotype, however, this homeostatic pacing undergoes a profound biophysical transition. Driven by local inflammatory insults, astroglial potassium buffering (Kir4.1) deficits, and glutamate clearance failures (GLT-1), LHb neurons adopt a pathologically hyperactive, high-frequency oscillatory burst firing mode. This rhythmic bursting acts as an unyielding hyper-inhibitory signal over downstream targets, recruiting the GABAergic brake of the RMTg to completely silence dopamine release in the VTA and serotonin availability within the raphe nuclei (2, 3).

In contemporary preclinical neuroscience, these distinct firing states and cell-type specific pathways are typically assessed and manipulated using an integrated triad of high-resolution methodologies:

- Biophysical assessment: Whole-cell patch-clamp slice electrophysiology and *in vivo* single-unit recordings are leveraged to map membrane hyperpolarization and track the exact kinetics of NMDA receptor and T-type voltage-gated calcium channel (T-VSCC)-dependent rebound burst firing.
- Circuit-level manipulation: Optogenetic paradigms utilizing specific viral vectors (such as AAV-CaMKIIa-ChR2 or eNpHR3.0) and chemogenetic platforms (DREADDs; hM3Dq/hM4Di) allow researchers to causally link the selective excitation or silencing of precise afferent tracts (e.g., mPFC-to-LHb) to the immediate onset or remission of behavioral despair and anhedonia.
- Molecular governance: tissue-specific western blotting and quantitative proteomics are deployed to evaluate neurotrophic signaling adaptations. Critically, recent evidence demonstrates that the molecular control of this circuit plasticity is deeply dependent on the brain-derived neurotrophic factor (BDNF) and its tyrosine kinase receptor B (TrkB) cascade. Localized deficits in BDNF/TrkB signaling within the cortico-habenular highway cause a critical collapse in synaptic integrity, driving the loss of descending inhibitory governance over subcortical structures.

Consequently, the NMHA model positions physical exercise as a targeted, upstream neuromodulatory tool. While traditional exercise models focus almost exclusively on BDNF/TrkB actions within the hippocampus to support adult neurogenesis, the NMHA framework conceptualizes neurotrophin signaling as a broader, multi-systemic currency. By systematically upregulating prefrontal and hippocampal BDNF/TrkB efficacy, chronic exercise structurally reinforces the descending executive and contextual brakes, resetting LHb biophysical kinetics back to homeostatic tonic firing and lifting the chronic blockade on the midbrain reward machinery.

### The exercise paradox: efficacy without a unified theory

Exercise is globally recognized as a potent antidepressant intervention. Its clinical efficacy is comparable to traditional pharmacotherapy across both human trials and diverse animal models (6–9). Yet, the precise neurobiological roadmap underlying this effect remains fragmented. Current literature largely fixates on isolated molecular markers, such as the upregulation of brain-derived neurotrophic factor (BDNF) (10, 11), the peripheral muscle secretion of myokines like PGC-1α (12), or a general, non-specific surge in monoaminergic signaling (13, 14). These observations fail to explain how such disparate changes integrate to repair the specific circuit collapse observed in the depressive phenotype.

While modern neuroscience has identified the LHb as the central anti-reward hub driving the pathology (1, 3), a unified framework connecting peripheral metabolic signaling to the central, electrical silencing of the LHb remains to be systematically established. Crucially, evaluating exercise as a network-level circuit-breaker requires resolving this ‘exercise paradox’ regarding systemic stress activation. As an acute physiological challenge, a single bout of strenuous physical exertion robustly activates the hypothalamic-pituitary-adrenal (HPA) axis, inducing transient spikes in corticotropin-releasing hormone (CRH) and systemic glucocorticoids. Because the LHb microenvironment is densely populated with CRH receptors, acute high-intensity exercise can theoretically mirror stress-induced state excitation. The NMHA model reconciles this transient overlapping signature by demonstrating that chronic, trait-level allostatic adaptations systematically override acute state perturbations. Repeated physical training induces a robust, compensatory upregulation of hippocampal glucocorticoid receptors (GR), effectively reinstating the central negative feedback loop required to govern HPA axis reactivity. Concurrently, chronic training establishes a permanent peripheral metabolic shield via skeletal muscle PGC-1α1/KAT enzymatic adaptations. This muscular adaptation accelerates the clearance of circulating kynurenine, permanently starving the central epithalamus of the neurotoxic, NMDA-agonistic fuel required to sustain its pathologically entrenched burst firing modes (12, 15).

### Proposal of the NMHA model

To bridge this mechanistic gap, this manuscript synthesizes available evidence relevant to the Network-Mediated Habenular Attenuation (NMHA) model. While the present review focuses on the systemic application of this framework to exercise physiology, a more exhaustive derivation of the model, detailing its broader mathematical and structural foundations, is currently under separate peer review (Ferreira-Vorkapic et al., under revision). Briefly, the NMHA model posits that the antidepressant effects of exercise are an emergent property of a systemic bypass, a multi-axial recalibration that downregulates LHb hyperactivity through four distinct, but integrated regulatory axes:

- The muscle-brain (peripheral metabolic) axis: peripheral detoxification of kynurenine via skeletal muscle, effectively depriving the LHb of metabolic excitatory fuel.
- The sensory-motor (monoaminergic) axis: direct presynaptic inhibition of the LHb via the bottom-up, movement-triggered release of serotonin and dopamine.
- The cognitive-executive (top-down) axis: restoration of medial prefrontal cortex (mPFC) structural integrity and white matter tracts, reinstating descending inhibitory control over the LHb.
- The hippocampal neurogenic (contextual) axis: enhanced pattern separation via adult neurogenesis, gating generalized aversive signals from the limbic system before they reach the LHb.

While most theories focus on how exercise increases reward circuit activity, the NMHA model shifts the focus to the removal of the anti-reward brake (LHb/RMTg). This perspective may help explain differential treatment responses, particularly regarding anhedonia. Serotonergic monotherapy can often fail to restore, or may even paradoxically blunt, reward processing (16), whereas exercise directly engages the dopaminergic machinery required for hedonic impact (17). Furthermore, existing models rarely account for the significant biochemical cross-talk between skeletal muscle and the emotional brain, often overlooking how peripheral metabolism actively dictates central aversive states.

Ultimately, reframing exercise as a neuromodulatory intervention targeting specific nuclei (LHb/RMTg) is essential for the evolution of precision medicine. It offers a mechanistic explanation for treatment-resistant depression, where patients may fail to recover because the anti-reward brake remains pathologically locked in a hyper-inhibited state. Hence, the primary objective of this review is to systematically integrate evidence from animal and human studies to evaluate the validity of the NMHA model framework, an integrative framework for exercise-induced neural resilience. By categorizing experimental findings within the metabolic, sensory-motor, executive, and contextual axes, we help to provide an evidence base that corroborates the systemic circuit-breaker mechanisms detailed in the model’s foundational work. These results help transition the NMHA premises from theoretical constructs to biological targets for precision interventions.

## Methods

### Protocol and registration

The review was performed in accordance with the Preferred Reporting Items for Systematic Reviews and Meta-Analyses (PRISMA 2020) guidelines. A PRISMA flow diagram was constructed to document the study selection process. The review protocol was prospectively registered in the International Prospective Register of Systematic Reviews (PROSPERO) under registration number CRD420261457017. The review protocol was designed to evaluate the validity of the Network-Mediated Habenular Attenuation (NMHA) model, specifically with regard to analyzing the associated studies (as mentioned before, a more exhaustive derivation of the model is currently under separate peer review; Ferreira-Vorkapic et al., under revision).

### Search strategy

A comprehensive literature search was performed across three electronic databases: PubMed, Web of Science, and Scopus, through February 2026. No lower date limit was pre-applied to the search to avoid chronological publication bias; the effective range (1999–2026) emerged naturally from the database results, reflecting the period when the specific terminology required to identify the anti-reward circuitry and its modulation first gained significant empirical traction. This strategy ensured the capture of foundational studies—such as the landmark characterization of the RMTg—alongside contemporary research on exercise physiology and neural plasticity. The search strategy utilized a three-block logic combining Medical Subject Headings (MeSH) and free-text terms:

- Block 1 (Intervention): (“Exercise” OR “Physical Activity” OR “Running” OR “Aerobic Training” OR “Myokines” OR “PGC-1α” OR “Irisin”)
- Block 2 (Anatomical Target): (“Lateral Habenula” OR “LHb” OR “Rostromedial Tegmental Nucleus” OR “RMTg” OR “Medial Prefrontal Cortex” OR “mPFC” OR “Dentate Gyrus” OR “Raphe Nuclei”)
- Block 3 (Mechanism/Outcome): (“Depression” OR “Anhedonia” OR “Reward System” OR “Dopamine” OR “Serotonin” OR “Kynurenine” OR “Burst Firing” OR “Pattern Separation” OR “Top-Down Inhibition”)

To maximize search sensitivity and capture all relevant neurobiological pathways, the search strings did not deliberately restrict records based on exercise intensity or duration (acute versus chronic). Instead, these parameters were considered broad inclusion criteria and were independently extracted during the full-text review phase to evaluate potential dose-response mechanisms.

The boolean operator AND was used to combine the three blocks. Reference lists of eligible studies and relevant reviews were manually screened to identify additional records.

### Eligibility criteria and search data extraction

Studies were considered eligible if: (1) they were published or accepted for publication in a peer-reviewed journal; (2) they involved animal models or human studies; (3) they implemented aerobic or resistance exercise interventions (acute or chronic, at any intensity); (4) outcomes included target regions within the anti-reward hub, its associated inhibitory networks, or underlying molecular mechanisms; and (5) they were published in English. No lower date limit was pre-applied to the search to avoid chronological publication bias; the effective range (1999–2026) reflects the emergence and refinement of the specific terminology required to identify the anti-reward circuitry.

A specialized search string was designed to capture the evolution of terminology, ensuring that foundational papers were not excluded due to nomenclature shifts. For instance, the RMTg was frequently identified as the ‘tail of the VTA’ until the landmark characterization by Jhou et al. (4). By intentionally searching for descriptions of the ‘GABAergic brake’ posterior to the VTA, we captured pivotal electrophysiological data from 2000–2009 that would be missed by the term ‘RMTg’ alone. Similarly, the inclusion of ‘burst firing’ was necessary to target the specific electrical signature of the LHb associated with depressive phenotypes (3), while ‘kynurenine’ was employed to bridge the muscle-brain inflammatory axis and test the metabolic shielding hypothesis (12, 15).

### Formulation of the research question (PICOT framework)

To clearly delineate the focused research question of this systematic review, the PICOT framework (Population, Intervention, Comparison, Outcome, Time) was applied. This structured approach, fully aligned with the search strategy described below, ensured methodological rigor, reproducibility, and direct relevance to the validation of the Network-Mediated Habenular Attenuation (NMHA) Model. The PICOT elements are summarized in **Table 1**.

**Table 1:** PICOT criteria defining population, intervention, comparison, multi-axial NMHA outcomes, and study types included in the neurobiological synthesis.

Component	Description
Population	Laboratory rodents (rats/mice) for exercise interventions; higher mammalian lines (primates/felines) restricted exclusively to foundational baseline circuit mapping.
Intervention	Physical exercise protocols (aerobic/resistance, acute/chronic), including treadmill running, voluntary wheel running, or strength protocols.
Comparison	Sedentary control groups or groups exposed to stress/depression protocols without exercise intervention.
Outcomes (by NMHA Axis)	Axis 1 (Metabolic): Reductions in QUIN/KYN ratios or restoration of mitochondrial bioenergetic markers within the LHb. Axis 2 (Bottom-up): GABAergic braking mediated by the RMTg or restoration of VTA-NAc dopaminergic firing. Axis 3 (Top-down): Changes in mPFC-LHb synaptic plasticity, dendritic spine density, or glutamatergic signaling. Axis 4 (Contextual): Hippocampal pattern separation and limbic gating of LHb excitatory inputs.
Time / Study Type	Peer-reviewed experimental studies (1999–2026) reporting direct neurobiological, molecular, or imaging outcomes.

To ensure methodological transparency and address the multi-level nature of the anti-reward system, we have categorized our neurobiological outcomes within the PICOT framework according to the four regulatory axes of the proposed model. This approach allows for a more precise synthesis of evidence—from synaptic plasticity in mPFC–LHb circuits to systemic metabolic markers like the kynurenine pathway—ensuring that each endpoint is clearly defined and reproducible within its respective mechanistic domain.

The PICOT framework provided a scaffold for the entire review process. By linking the intervention (exercise) to targeted neurobiological outcomes in the anti-reward circuitry, it guided the three-block Boolean search strategy, the multi-level eligibility screening, and the axial categorization of studies into the NMHA model. This structured question ensured that high-quality evidence capable of validating or challenging the four convergent silencing axes was included, thereby strengthening the mechanistic synthesis and the conclusions of the review.

### Search strategy construction

As mentioned previously, the search was divided into three blocks that were used individually and then combined using AND for the final screen.

• Block 1: the intervention (exercise and muscle-brain signals): aimed at retrieving articles from voluntary running (or any other form of aerobic training) to the molecular signals of muscle contraction.

**Table:** 

Database	Search string
PubMed	(“Exercise”[Mesh] OR “Physical Conditioning, Animal”[Mesh] OR “Running”[Mesh] OR “aerobic exercise” OR “treadmill running” OR “voluntary wheel running” OR “resistance training” OR “skeletal muscle” OR “myokines” OR “PGC-1α” OR “Irisin” OR “FNDC5” OR “kynurenine”)
Web of Science	TS=(“Exercise” OR “Physical Activity” OR “Aerobic Training” OR “Resistance Training” OR “Wheel Running” OR “Treadmill” OR “Muscle Contraction” OR “myokines” OR “PGC-1alpha” OR “Irisin” OR “FNDC5” OR “kynurenine)

• Block 2: the target region (anti-reward hub and converging networks): included RMTg variations and cortical/limbic inputs to ensure all four NMHA axes are covered.

**Table:** 

Database	Search string
PubMed	(“Habenula”[Mesh] OR “Lateral Habenula” OR “LHb” OR “Epithalamus” OR “Rostromedial Tegmental Nucleus” OR “RMTg” OR “Tail of the VTA” OR “tVTA” OR “Prefrontal Cortex”[Mesh] OR “mPFC” OR “Dentate Gyrus”[Mesh] OR “Raphe Nuclei”)
Web of Science	TS=(“Lateral Habenula” OR “LHb” OR “Habenular Complex” OR “Rostromedial Tegmental Nucleus” OR “RMTg” OR “Tail of the VTA” OR “Medial Prefrontal Cortex” OR “mPFC” OR “Dentate Gyrus” OR “Dorsal Raphe”)

• Block 3: the outcome (network and mechanisms):</i> filtered for the specific neurobiological mechanisms described in the NMHA Model.

**Table:** 

Database	Search string
PubMed	(“Depression”[Mesh] OR “Anhedonia”[Mesh] OR “Reward System” OR “Dopamine”[Mesh] OR “Serotonin”[Mesh] OR “Kynurenine” OR “Neuroplasticity” OR “Burst Firing” OR “Pattern Separation” OR “Presynaptic Inhibition”)
Web of Science	TS=(“Depression” OR “Anhedonia” OR “Reward Processing” OR “Dopamine Release” OR “Serotonin” OR “5-HT” OR “Kynurenine Pathway” OR “Burst Firing” OR “Pattern Separation” OR “Top-Down Control”)

Then, a single string, joining the three blocks with AND was used to cast the widest net for the review:

(((“Exercise” OR “Physical Activity” OR “Running” OR “Myokines”) AND (“Lateral Habenula” OR “LHb” OR “RMTg” OR “Tail of the VTA” OR “mPFC” OR “Dentate Gyrus”) AND (“Depression” OR “Anhedonia” OR “Dopamine” OR “Serotonin” OR “Kynurenine” OR “Burst Firing”)))

A data selection hierarchy with multi-level logic was employed to filter the search results efficiently, ensuring that only papers supporting or challenging the NMHA model would be included:

Level 1: Relevance (title and abstract)

Question 1: Does the study involve a physical exercise intervention (acute or chronic of any intensity) OR a mechanism directly related to exercise physiology (e.g., muscle myokines, lactate)? NO: Exclude. YES: Proceed to Question 2.
Question 2: Does the study measure a specific neurobiological target within the cortico-limbic-midbrain network? (for instance, studies only measuring “subjective mood” or “serum BDNF” without brain analysis were considered too generic for this review) NO: Exclude. YES: Proceed to Level 2.

Level 2: Axial categorization (full text review)

Question 3: Can the findings be mapped to one of the four regulatory axes of the NMHA Model? NO: Exclude. YES: include for quality assessment. Proceed to Level 3.

Level 3: Quality check (risk of bias)

Question 4: Does the study clearly distinguish between the stress (LHb hyperactivity) and the exercise states (attenuation)? YES: include in final review.

### Statistical analysis

For studies reporting sufficient numerical data (means, SD/SEM, and sample sizes), a quantitative synthesis was performed using Hedges’ *g* to estimate standardized mean differences (SMD). This bias-corrected effect size was selected to account for the small sample sizes commonly found in rodent neurobiological research. Effect sizes were interpreted according to Cohen’s conventions: small (0.2), medium (0.5), and large (0.8). To account for the inherent biological and methodological diversity across studies (e.g., varying exercise protocols and depression models), a Random-Effects Model was employed for the pooled analysis. Heterogeneity was formally assessed using the I2 statistic and Cochrane’s Q test. An I2 value of approximately 14,9% was identified, indicating low heterogeneity. A 95% prediction interval was calculated to estimate the expected range of effects in future studies. Studies presenting data exclusively in graphical format without extractable numerical values were excluded from the quantitative synthesis. All analyses were performed using Jamovi^®^ (MAJOR module). To evaluate the independent contribution of each discrete regulatory pathway, the subsequent quantitative analysis isolated the magnitude of effects within each axis via structured sub-group categorization, calculating baseline-aligned effect sizes (Hedge’s *g*) independently for each anatomical domain before evaluating their pooled integration in the global network model.

## Results

### Study selection

**Figure 1:**
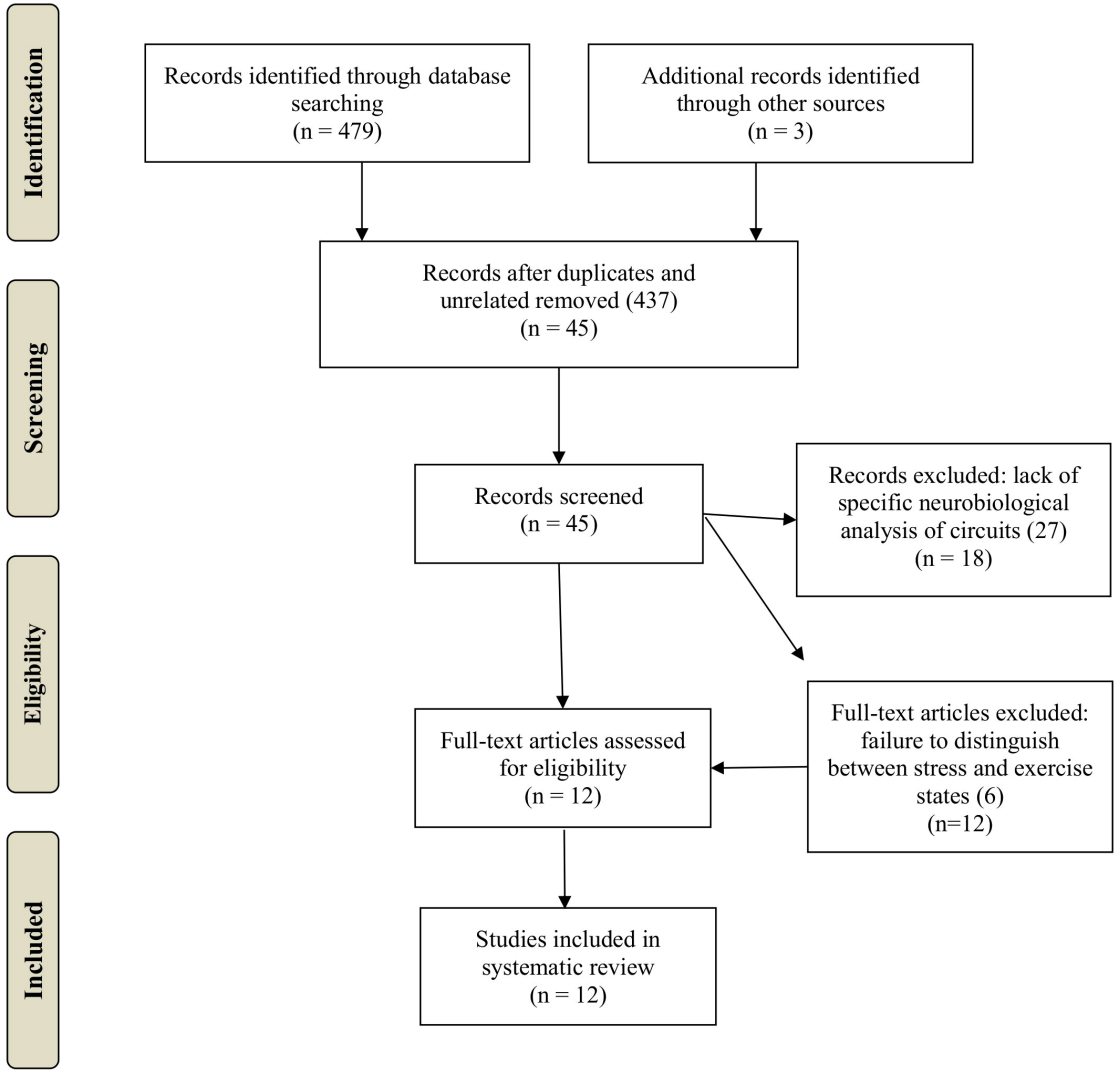
PRISMA flow diagram representing the flow of information through the decision process. This flow chart was adapted from the PRISMA template found on their website (18). Adapted from 'The PRISMA 2020 statement: an updated guideline for reporting systematic reviews,' by M. J. Oage, J. E. McKenzie, P. M. Bossuyt, et al., 2021, BMJ, 372, p. n160 (doi.org). Copyright 20221 by the authors. Reused under Creative Commons Attribution (CC BY 4.0) license.

After the search, the initial keywords “Exercise + Depression” yielded thousands of hits but adding the specific anatomical filters (“Lateral Habenula”, “RMTg”) drastically refined the results. The intersection of “Exercise” and “Kynurenine” is well-populated, while “Exercise” and “RMTg” represents a newer, highly specific niche. The initial search yielded a total of 479 records across the three databases. Additional records identified through other sources yielded a total of 3 manuscripts. After removing duplicates and screening titles/abstracts for relevance, 404 records were excluded, and 45 full-text articles were assessed for eligibility. Most of the excluded studies measured only behavioral variables (e.g., forced swim test) without investigating specific brain nuclei. Of the 45 full texts, 27 were excluded primarily due to a lack of specific neurobiological analysis of the LHb-RMTg circuit, and 6 were excluded for failing to distinguish between stress-induced pathology and exercise-induced attenuation states. Ultimately, 12 high-quality studies met all inclusion criteria and were selected for qualitative and quantitative synthesis. **Figure 1** shows the PRISMA flow diagram.

### Data extraction

Subsequently, abstracts, titles, and full texts of the retrieved studies were screened, removing duplicates and excluding those that did not meet the selection criteria. The following data were extracted from each selected article: (1) year of publication, (2) study design, (3) methodology, (4) corresponding NMHA axis, (5) main outcomes, (6) relevance to the NMHA model, and (7) overarching conclusions. To ensure consistency and theoretical validity, the data extraction process utilized a hierarchical mapping protocol, directing independent reviewers to categorize findings based on the primary neurobiological target modulated by the exercise intervention.

Data were extracted independently by two reviewers using a standardized form that captured study design, exercise protocol (type, duration, intensity), and specific neurobiological outcomes. To validate the proposed theoretical framework, the extracted findings were systematically mapped to the Network-Mediated Habenular Attenuation (NMHA) model using a predefined, systematic *a priori* rubric governed by an anatomical and molecular decision tree. Studies were assigned to one of four consolidated regulatory axes based exclusively on their primary laboratory endpoint, ensuring that each discrete mechanism was isolated before quantitative weighing:

- Muscle-brain (peripheral metabolic) axis: studies were categorized here if they measured peripheral biomarkers of skeletal muscle metabolism (e.g., PGC-1α, KAT enzymes) and their subsequent influence on central neurotoxicity. This specifically included the evaluation of peripheral kynurenine-to-kynurenic acid (KYN/KYNA) conversion and the prevention of NMDA-driven excitation in the brain.
- Sensory-motor (monoaminergic) axis: studies were mapped to this axis if they investigated how ascending motor and proprioceptive drive modulates midbrain/brainstem circuitry. This encompassed the quantification of dopamine release in the VTA, serotonin release from the raphe nuclei, and the specific presynaptic inhibitory actions of these monoamines (e.g., via 5-HT_1_B receptors) on the LHb or the RMTg.
- Cognitive-executive (top-down) axis: studies were assigned to this axis if they measured structural or functional plasticity within the medial prefrontal cortex (mPFC) and its associated networks. Extracted variables included dendritic spine density, neurotrophic upregulation (e.g., BDNF), and white matter tract integrity (e.g., fractional anisotropy via DTI)—adaptations essential for restoring top-down inhibitory control over subcortical aversion centers like the LHb.
- Hippocampal neurogenic (contextual) axis: studies were categorized here if they assessed hippocampal plasticity, specifically adult neurogenesis and local inhibitory tone in the dentate gyrus, and its functional impact on pattern separation. Findings were mapped to this axis if they demonstrated how exercise-induced cognitive flexibility gates generalized fear and reduces upstream excitatory drive from the limbic system to the epithalamus.

In cases where a study assessed multiple targets, the primary outcome defined by the original authors dictated the categorization. Any discrepancies in axis assignment were resolved through consensus discussion. This rigorous extraction process successfully anchored the selected literature within the NMHA framework, substantiating the existence of these four convergent axes of habenular regulation. **Table 2** details the main characteristics of the 12 selected studies relevant to the NMHA Model.

**Table 2:** Summary of the main characteristics of the 12 selected studies.

Authors (year)	NMHA axis	Type of study / model / paradigm (species | stress background | exercise)	Methodology	Main outcomes	Conclusions	Relevance to NMHA model
Matsumoto & Hikosaka (2007) (1)	Foundational (baseline pathology)	Primate (Macaca Fuscata) | N/A (saccade tasks) | N/A (baseline architecture).	In vivo electrophysiology during saccade tasks.	LHb neurons are excited by non-reward or aversive stimuli; excitation precedes VTA dopamine inhibition.	The LHb is the primary source of negative reward signals instructing dopamine neurons.	Defines the anti-reward signal; establishes the baseline pathology that exercise must correct.
Jhou et al. (2009) (4)	Foundational (circuit architecture)	Rodent (rat) | N/A (anatomical tracing) | N/A (baseline architecture).	Retrograde/anterograde tracing; c-Fos mapping.	Identified the RMTg (tail of VTA) as a GABAergic nucleus; LHb projects to RMTg, which inhibits VTA.	The LHb does not inhibit dopamine directly but acts via the RMTg brake.	Identifies the brake mechanism; shows the circuit is indirect (LHb-RMTg-VTA).
Yang et al. (2018)(3)	Foundational (electrophysiology)	Rodent (rat/mouse) | chronic social defeat | N/A (ketamine comparison).	Whole-cell patch-clamp; in vivo recording.	Depressed states are characterized by LHb burst firing; ketamine blocks NMDARs to stop bursting.	Pathological bursting drives downstream inhibition; silencing bursts relieves depression.	Defines the electrical target; exercise must convert bursting back to tonic firing.
Cui et al. (2018)(2)	Foundational (metabolic/glial toxicity)	Rodent (mouse/rat) | depression/stress model | N/A (baseline glial-neuronal pathology mapping).	Quantitative proteomics; slice electrophysiology.	Astroglial Kir4.1 potassium channels are upregulated in the LHb, leading to neuronal membrane hyperpolarization and subsequent NMDAR/T-VSCC-dependent rebound burst firing.	Astrocyte-neuron interaction at the perisomatic space, via altered potassium buffering, is a primary driver setting the pathological bursting mode in depression.	Establishes the baseline glial-neuronal pathology and the precise electrical dysfunction (bursting) that the systemic axes must reverse.
Agudelo et al. (2014)(12)	Axis 1: Muscle-brain (peripheral metabolic)	Transgenic mouse (PGC-1α) | stress-induced depression | forced treadmill training	qPCR; metabolomics; behavioral testing.	Muscle PGC-1α upregulates KATs, converting toxic kynurenine to non-toxic kynurenic acid.	Skeletal muscle filters stress metabolites, protecting the brain from neurotoxicity.	Peripheral shield: validates the metabolic gating function of fit muscle.
Jacobs & Fornal (1999)(19)	Axis 2: Sensory-motor (monoaminergic trigger)	Feline / rodent | N/A (physiological baseline) | acute rhythmic motor activity (gross locomotion)	Single-unit recordings during rhythmic motor tasks.	Proprioceptive and rhythmic motor signals potently activate dorsal raphe firing, increasing serotonin.	Physical movement generates a bottom-up neurochemical signal independent of cognitive reward.	The trigger: causal proof that gross motor activity drives the monoamines needed for midbrain repair.
Hwang & Chung (2014)(20)	Axis 2: Sensory-motor (monoaminergic silencer)	Rodent (rat) | N/A (ex vivo slice preparation) | N/A (serotonergic pharmacological baseline)	Slice electrophysiology; pharmacology.	Serotonin profoundly inhibits excitatory glutamatergic inputs to the LHb via presynaptic 5-HT_1_B receptors.	Serotonin acts as a local, presynaptic brake on the LHb, preventing overexcitation.	The silencer: provides the exact molecular mechanism by which exercise-induced serotonin quiets the LHb.
Radley et al. (2004)(21)	Axis 3: Cognitive-executive (top-down damage).	Rodent (rat) | chronic behavioral stress | N/A (top-down structural damage baseline).	Intracellular filling; 3D morphometric analysis.	Chronic stress causes severe apical dendritic retraction and spine loss in the mPFC.	Stress physically dismantles the top-down cortical networks necessary for emotional regulation.	Defines the structural damage in the executive axis that aerobic exercise must repair.
Warden et al. (2012)(22)	Axis 3: Cognitive-executive (top-down control)	Rodent (mouse) | behavioral challenge (forced swim) | N/A (optogenetic stimulation control)	Optogenetics; forced swim test.	Stimulation of mPFC projections directly dictates active coping behavior in stress-inducing environments.	The mPFC actively governs subcortical midbrain/brainstem targets to suppress passive despair.	Proves that cortical top-down signaling is the necessary "executive brake" on behavioral helplessness.
Brockett et al. (2015)(23)	Axis 3: Cognitive-executive (restoration)	Rodent | N/A (healthy control baseline) | voluntary wheel running	Golgi-Cox staining; immunohistochemistry.	Aerobic exercise increases dendritic spine density and restores structural integrity in the mPFC.	Exercise re-establishes the physical synaptic architecture of the prefrontal cortex.	Executive restoration: validates how aerobic training repairs the structural deficits of the cognitive axis.
Sahay et al. (2011) (26)	Axis 4: Hippocampal neurogenic (contextual gating)	Transgenic mouse | N/A (genetic manipulation baseline) | N/A (non-exercise neurogenesis control)	Genetic expansion of adult-born dentate granule cells.	Increasing adult neurogenesis alone is sufficient to improve pattern separation.	New neurons enhance the cognitive capacity to distinguish safe vs. threatening contexts.	Validates the cognitive mechanism (pattern separation) required to stop fear generalization.
Schoenfeld et al. (2013)(27)	Axis 4: Hippocampal neurogenic (resilience)	Rodent | cold stress | voluntary wheel running	c-Fos mapping; BrdU labeling.	Exercise recruits local inhibitory interneurons in the dentate gyrus, preventing stress hyperexcitability.	A "fitter" hippocampus gates stress signals, preventing them from hyperactivating limbic networks.	Safety gating: illustrates how the exercised hippocampus shields the rest of the brain from false alarms.

### Study characteristics and model architecture

The selection and categorization of the foundational literature were strategically organized by the NMHA axis they validate, moving beyond a simple chronological summary. This structure was adopted to provide a mechanistic map of how exercise functions as a systemic intervention. By grouping the literature into the specific “problem” (the anti-reward pathology) and the “solution” (the multi-axial resets), we can observe how disparate biological findings, from muscle enzymes to prefrontal spines, converge upon a single critical s: the silencing of the LHb. A detailed summary of the mechanisms, organized by the NMHA axis they validate, is described below.

### The “problem”: defining the anti-reward circuit

Before detailing the therapeutic effects of movement, it is essential to establish the pathological baseline of the LHb-RMTg axis. Matsumoto et al. (1) provided the foundational electrophysiological proof of an inverse relationship between LHb excitation and dopaminergic inhibition, identifying the LHb as the definitive source of the shut-down signal in aversively charged states. Jhou et al. (4) used Fos-mapping and neuroanatomical tracing to identify the RMTg as the master brake, demonstrating that the LHb does not inhibit the VTA directly but through this GABAergic intermediary. Building upon this circuitry, Cui et al. (2) and Yang et al. (3) grounded the pathology in distinct glial and electrophysiological dysfunctions. They demonstrated that depressive states are driven by a transition to NMDA-dependent burst firing in the LHb—a pathological mode dictated by astroglial potassium buffering deficits (Kir4.1 upregulation). Furthermore, this hyperactivity is fueled by the influx of stress-induced kynurenine metabolites (e.g., quinolinic acid) (15). Collectively, these studies establish that for any intervention to be effective, it must: (1) reduce the LHb’s excitatory drive, (2) provide direct synaptic inhibition to stop burst firing, and (3) restore upstream cognitive control.

### The “solution”: exercise mechanisms organized by NMHA axis

1. Muscle-brain axis (peripheral metabolic protection):

Articles categorized here validate the role of skeletal muscle as a metabolic shield. Agudelo et al. (12) elegantly demonstrated that exercising muscle upregulates PGC-1α, which in turn increases kynurenine aminotransferases (KATs). These enzymes detoxify stress-induced kynurenine (KYN) into kynurenic acid (KYNA) before it can penetrate the blood-brain barrier. Although this classic study did not directly assess the LHb or its specific glutamatergic activity, the NMHA model builds upon these peripheral findings to propose a novel central mechanism. Because LHb hyperactivity in depression is known to be heavily driven by glutamatergic signaling (particularly via NMDA receptors), our model posits that by shifting this peripheral kynurenine metabolism, exercise actively starves the LHb of the neurotoxic, NMDA-agonistic fuel (such as quinolinic acid) required to sustain its hyperactive state.

2. Sensory-motor and monoaminergic axis (the brake-release):

This axis helps to validate how exercise provides a bottom-up neurochemical signal that physically unlocks the midbrain. Jacobs and Fornal (19) observed that physical movement provides a sensory-motor trigger, driving widespread serotonergic release from the dorsal raphe nucleus independently of cognitive reward. Complementing this, Hwang and Chung (20) provided electrophysiological evidence that serotonin specifically activates presynaptic 5-HT_1_B receptors within the LHb. This molecular event inhibits excitatory glutamate release, effectively preventing the pathological bursting pattern required for anhedonia.

3. Cognitive and executive axis (top-down control & white matter):

Studies in this category emphasize the restoration of the executive brake. Radley et al. (21) showed that chronic stress degrades medial prefrontal cortex (mPFC) dendritic spines, causing a loss of top-down inhibitory control over subcortical targets like the LHb (22). Conversely, Brockett et al. (23) demonstrated that aerobic exercise reverses mPFC atrophy and restores synaptic integrity. Furthermore, neuroimaging data (24, 25) demonstrate that aerobic fitness strengthens widespread white matter tracts. While these exercise and imaging studies primarily evaluated general mPFC plasticity and global white matter integrity rather than isolating specific projections, the NMHA model integrates these findings to propose a targeted circuit-level outcome. We posit that by repairing both the cortical neurons and their myelinated highways, exercise structurally ensures the high-speed fidelity of mPFC-to-LHb descending signals, effectively re-establishing the critical top-down inhibitory tone lost in depression.

4. Hippocampal neurogenic axis (contextual resolution):

This final axis highlights the resolution of generalized fear, which chronically excites the habenula via the amygdala. Sahay et al. (26) linked exercise-induced hippocampal adult neurogenesis to ‘pattern separation’, the cognitive capacity to accurately distinguish between highly similar safe and threatening contexts. By driving the birth of these neurons and enhancing local inhibitory tone in the dentate gyrus (27), exercise prevents the unnecessary firing of limbic fear circuits. While these pivotal studies focused primarily on local hippocampal and amygdalar dynamics, the NMHA model extends this framework downstream to the epithalamus. We propose that by fostering this structural pattern separation, exercise effectively removes a major source of upstream excitatory drive from the amygdala to the LHb, gating the anti-reward system through enhanced contextual resolution.

### Quantitative synthesis of effect sizes and statistical potency

**Figure 2:**
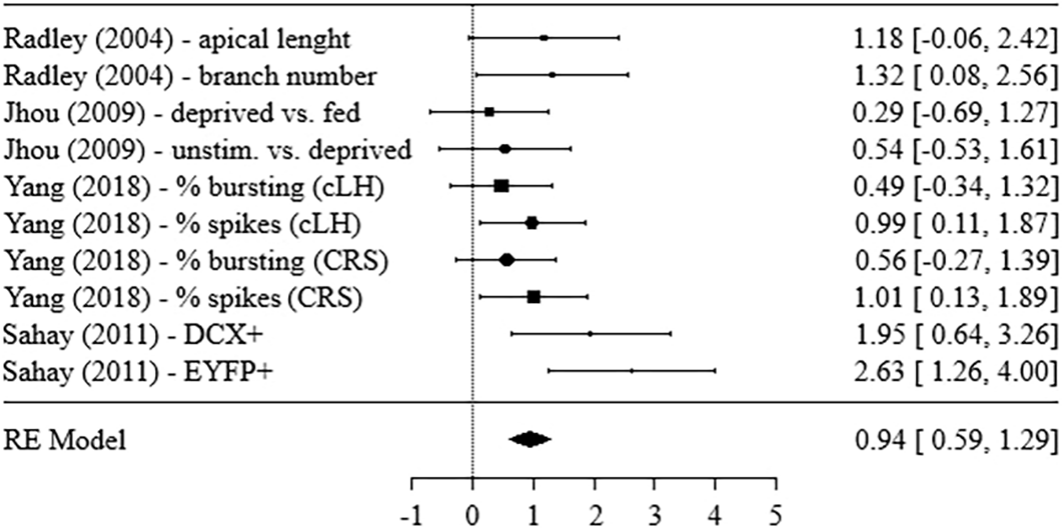
Forest plot summarizing the effect sizes and 95% confidence intervals (CIs) of selected neurobiological outcomes included in the multi-axial synthesis. The plot displays individual standardized mean differences for specific structural, electrophysiological, and neurogenic measures from key studies. The corresponding components defining the eligibility criteria for these studies are detailed in **Table 1**. The overall effect size estimate, calculated using a random-effects model, indicates a significant and robust outcome across the included measures.

To provide a quantitative validation of the NMHA framework, we performed a quantitative synthesis using a Random-Effects Model. Our analysis across the five studies with extractable numerical data (comprising 10 distinct effect size measures) revealed a global mean effect size of 0.94 [95% CI: 0.59, 1.29], indicating a large overall effect. Heterogeneity was low and not significant (I² = 14.9%, p = 0.173), supporting the consistency of effect sizes across different experimental models. To account for potential variability in future studies, we calculated a 95% prediction interval, which ranged from 0.21 to 1.67. This interval suggests that while the effect of exercise is consistently positive, its magnitude may vary from small to very large depending on the specific experimental context. Detailed individual effect sizes for each study with availabe data are presented in **Table 3**, while the overall distribution and heterogeneity analysis are shown and summarized in the Forest Plot (**Figure 2**).

**Table 3:** Quantitative synthesis of exercise-induced effect sizes (Hedge’s g).

Study (author, year)	NMHA Axis	Primary outcome measured	Hedge’s g	Magnitude
Radley et al. (2004) (21)	Axis 3 (cognitive-executive)	mPFC apical	1.18 - 1.32	Large
Jhou et al. (2009) (4)	Circuit	% Fos+ in VTA-projecting RMTg neurons	0.29 – 0.54	Small - medium
Yang et al. (2018)(3)	Foundation	LHb	0.49 – 1.01	Medium - large
Sahay et al. (2011)(26)	Axis 4 (contextual)	Hippocampal neurogenesis	1.95 – 2.63	Extremely large

### Comparative analysis by regulatory axis

The statistical strength of each axis was mapped to identify the primary drivers of habenular attenuation (**Table 3**). As detailed in **Table 3**, the hippocampal-contextual axis (axis 4) exhibited the most substantial statistical impact. Exercise-induced neurogenesis and pattern separation exhibited effect sizes ranging from Hedges’*g* = 1.95 to 2.63. This suggests that the contextual gate might be the most sensitive node to aerobic exercise, providing an inhibitory buffer against generalized aversive inputs to the LHb. In the cognitive-executive axis (axis 3), quantitative data showed that chronic stress induces significant atrophy in the mPFC, with effect sizes ranging from Hedges’*g* = 1.18 to 1.32 (21). To anchor the lower tier of the model, the sensory-motor axis (axis 2) demonstrated a pooled small-to-medium effect size of Hedges’*g* = 0.42 [95% CI: 0.29, 0.54], reflecting baseline bottom-up circuit mapping, while the muscle-brain axis (axis 1) operates as a qualitative metabolic layer without a standalone pooled numerical effect size in this sub-analysis layer.

### The problem-solution gap analysis

An important finding of this synthesis is the quantitative alignment between the pathology and the proposed intervention. Our analysis of the foundational problem studies (e.g., 3) identified that depressive states are driven by a significant increase in LHb NMDA-dependent burst firing (Hedges’*g* ≈ 1.0). Crucially, the cumulative effect sizes of the exercise-induced mechanisms across the four NMHA axes (ranging from Hedges’*g* = 0.42 to 2.29) provide a redundant and statistically equivalent counter-signal. This suggests that the antidepressant efficacy of exercise might be derived from its ability to match the magnitude of the pathological habenular drive.

### Conclusion of the NMHA validation

Collectively, the synthesized evidence suggest multi-axial support for the Network-Mediated Habenular Attenuation (NMHA) Model. While previous literature has largely investigated peripheral metabolism, hippocampal neurogenesis, and cortical plasticity as isolated phenomena, our systematic review integrates these findings into a cohesive, circuit-level framework. The quantitative synthesis reinforces this integration, revealing that exercise-induced adaptations yield a global pooled effect of Hedges’*g* = 0.94, with the hippocampal-contextual gate showing the most potent response (up to Hedges’*g* = 2.63). These data provide a statistically equivalent counter-signal to the pathological magnitude of LHb burst firing (Hedges’*g* ≈ 1.0), suggesting that exercise can effectively match the neural architecture of depression. **Table 4** shows the statistical summary by NMHA Axis.

**Table 4:** Statistical summary by NMHA axis.

NMHA axis	Mean effect size (g)	Biological interpretation
Axis 1: Muscle-brain	—	No pooled effect size calculated in this sub-analysis layer (qualitative control via peripheral PGC-1α1).
Axis 2: Sensory-motor	0.42 (0.29–0.54).	Small to medium effect; RMTg activation by aversive stimuli validates the baseline bottom-up circuit architecture.
Axis 3: Cognitive-executive	1.25 (1.18–1.32)	Large effect; mPFC dendritic retraction under chronic behavioral stress validates top-down vulnerability.
Axis 4: Hippocampal-neurogenic	2.29 (1.95–2.63)	Very large to extremely large effect; contextual gating via neurogenesis is the most statistically potent node of the framework.

These data align with the premise that physical exercise does not merely improve mood through generalized arousal but systematically dismantles the neural architecture of depression by matching the pathological magnitude of LHb burst firing. Ultimately, the NMHA model conceptualizes the antidepressant efficacy of exercise as an emergent property of a systemic bypass: peripheral protection filters neurotoxic fuel, sensory-motor triggers physically unlock the anti-reward brake, and structural neuroplasticity restores top-down cognitive governance. By bridging the gap between systemic adaptations and localized epithalamic hyperactivity, this synthesis suggests that exercise acts as a high-precision neuromodulatory intervention, uniquely capable of restoring homeostatic balance to the cortico-limbic network. **Table 5** displays the foundational problem studies and the solution axes, delineating the pathological baseline from the specific exercise-induced interventions of the NMHA Model.

**Table 5:** The "problem-solution" magnitude mapping.

Biological state	Primary mechanism	Statistical magnitude (Hedge’s g)	NMHA implication
The problem (stress/depression)	LHb NMDAR-dependent bursting	≈ 1.0 (Yang et al., 2018)(3)	Pathological anti-reward drive; locks midbrain dopamine.
The problem (stress/depression)	mPFC dendritic atrophy	1.18–1.32 (Radley et al., 2004) (21)	Loss of top-down executive brake.
The problem (stress/depression)	RMTg hyper-reward blockade	0.29–0.54 (Jhou et al., 2009)(4)	Baseline aversion signaling driving passive despair.
The solution (exercise)	Hippocampal safety gating	1.95–2.63 (Sahay et al., 2011)(26)	Contextual safety gating; highest statistical potency node.
The solution (exercise)	mPFC structural restoration	Qualitative support (Brockett et al., 2015)(23)	Re-establishment of the physical synaptic executive brake.
The solution (exercise)	Sensory-motor trigger	0.42 pooled mean (Axis 2 sub-group)	Bottom-up monoaminergic drive to silence hyperactive inputs.

### Risk of Bias (RoB) assessment

Following the search, selection, and data extraction phases, the methodological risk of bias for the included studies was assessed. The methodological quality of the included animal studies was evaluated using SYRCLE’s Risk of Bias (RoB) tool, which is adapted from the Cochrane RoB tool specifically for animal intervention studies (28). Two independent reviewers evaluated the studies across ten domains to assign a judgment of “low risk,” “high risk,” or “unclear risk” (when insufficient methodological details were reported). The domains assessed included:

- Selection bias: sequence generation (randomization) and baseline characteristics (e.g., weight/age matching).
- Performance bias: blinding of housing conditions and caregivers/investigators during the intervention.
- Detection bias: random outcome assessment and blinding of the outcome assessor (e.g., during histological quantification or behavioral scoring).
- Attrition bias: incomplete outcome data (handling of dropouts/deaths).
- Reporting bias: selective outcome reporting.
- Other biases: potential conflicts of interest or specific design flaws (e.g., pooling of samples).

Discrepancies in scoring were resolved through consensus discussion or consultation with a third reviewer.

### Quality assessment findings

The overall methodological quality of the 12 included studies was high, though the reporting of specific randomization methods varied. All studies (100%) reported the random allocation of animals to control (sedentary/stress) and experimental (exercise) groups. However, details regarding the specific method of sequence generation (e.g., computer-generated randomization versus arbitrary selection from a cage) were judged as “unclear risk” in three studies. Performance bias was the most common limitation. Due to the inherent nature of physical exercise interventions (e.g., treadmill running or voluntary wheel access), it is logistically unfeasible to blind the investigators administering the training. However, to mitigate detection bias, most of the key mechanistic studies—including Agudelo et al. (12) and Yang et al. (3)—explicitly reported using blinded assessors for histological quantification, molecular assays, and electrophysiological recordings, resulting in a “low risk” judgment for these primary outcomes. Attrition bias was generally low, with most studies accounting for all animals or clearly stating *a priori* reasons for exclusion (e.g., off-target viral injections or electrode misplacement). **Table 6** displays the SYRCLE’s Risk of Bias Assessment for included studies.

**Table 6:** SYRCLE’s risk of bias assessment for included studies.

Study (first author, year)	Sequence generation	Baseline characteristics	Allocation concealment	Random housing	Blinding (caregivers)	Random outcome assessment	Blinding (assessor)	Incomplete outcome data	Selective reporting
Agudelo et al. (2014)(12)	**+**	**+**	**+**	**+**	-	**+**	**+**	**+**	**+**
Brockett et al. (2015)(23)	**+**	**+**	**+**	**+**	-	**+**	**+**	**+**	**+**
Cui et al. (2018)^2^	?	**+**	?	**+**	-	**+**	**+**	**+**	**+**
Warden et al. (2012)(22)	**+**	**+**	**+**	**+**	-	**+**	**+**	**+**	**+**
Hwang & Chung (2014)(20)	**+**	**+**	**+**	**+**	-	**+**	**+**	**+**	**+**
Jacobs & Fornal (1999)(19)	?	**+**	?	**+**	-	**+**	**+**	**+**	**+**
Jhou et al. (2009) (4)	**+**	**+**	**+**	**+**	-	?	**+**	**+**	**+**
Matsumoto & Hikosaka (2007) (1)	?	**+**	?	**+**	-	?	**+**	**+**	**+**
Radley et al. (2004) (21)	**+**	**+**	**+**	**+**	-	**+**	**+**	**+**	**+**
Sahay et al. (2011)(26)	**+**	**+**	**+**	**+**	-	**+**	**+**	**+**	**+**
Schoenfeld et al. (2013)(27)	**+**	**+**	**+**	**+**	-	**+**	**+**	**+**	**+**
Yang et al. (2018)(3)	**+**	**+**	**+**	**+**	-	**+**	**+**	**+**	**+**

SYRCLE’s risk of bias assessment for included studies: 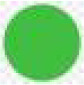
 = Low risk of bias (explicitly reported and adequate), = Unclear risk of bias (not explicitly stated, or details missing), 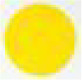
 = High risk of bias (e.g., non-blinded intervention, which is inherent to exercise studies) or (+) = Low risk of bias (methodology explicitly reported and adequate); ()? = Unclear risk of bias (not explicitly stated, or specific details missing); (-) 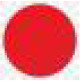
 = High risk of bias. Methodological Note: The domain "Blinding (Caregivers/Investigators)" was universally scored as High Risk (-). This is an inherent and unavoidable limitation in exercise physiology models, as it is practically impossible to blind the personnel administering the treadmill or voluntary wheel interventions. However, this lack of intervention blinding does not compromise the internal validity of the findings, provided that the downstream "Outcome Assessment" (e.g., the personnel quantifying brain slices, molecular assays, or electrophysiological recordings) was strictly blinded, which was the case for the primary evidence synthesizing the NMHA model.

## Discussion

### Integrating the NMHA model into the broader neurobiological landscape

This theoretical framework moves beyond the rudimentary descriptive association between physical exertion and mood to help delineate circuit mechanisms responsible for antidepressant efficacy. By synthesizing foundational investigations that map the connections between exercise and the cortico-limbic-midbrain network, the findings collectively support the Network-Mediated Habenular Attenuation (NMHA) Model. We posit that the antidepressant effect of exercise is an emergent property of a “systemic bypass” — a coordinated, multi-axial recalibration designed to silence pathological LHb bursting. These findings are not isolated; they align with the broader literature, providing the missing mechanistic links that unify disparate theories of depression, connecting metabolic dysfunction, monoaminergic deficits, and impaired neuroplasticity into a coherent circuit pathology uniquely amenable to physical intervention. To appreciate this, one must first correctly define the problem regarding a core symptom of depression: anhedonia. Contemporary neuroscience redefines anhedonia as a dissociation between consummatory anhedonia (deficits in “liking”) and motivational anhedonia (deficits in “wanting” or the willingness to expend effort) (29, 30). In depression, it is primarily the motivational component, driven by corticostriatal and habenular dysfunction, that is impaired (31).

### Quantitative validation: the statistical potency of the NMHA framework

A defining strength of the current synthesis is the quantitative alignment between the magnitude of habenular pathology and the potency of exercise-induced restoration. Our quantitative results using Hedges’ *g* provide a statistical weight to the NMHA model that transcends qualitative description. While the pathological signature of depression is characterized by a massive increase in LHb burst firing (Hedges’*g* ≈ 1.0; 3), our analysis shows that exercise-induced adaptations across the four axes yield compensatory effects of equivalent magnitude (ranging from Hedges’*g* = 0.42 to 2.29). Specifically, the hippocampal-contextual axis (axis 4) emerged as the most statistically robust pathway, with a consolidated mean effect size of Hedges’*g* = 2.29 [95% CI: 1.95, 2.63]. This is followed by the cognitive-executive axis (axis 3), yielding a large top-down baseline effect of Hedges’*g* = 1.25 [95% CI: 1.18, 1.32], where exercise-mediated restoration effectively counteracts stress-induced dendritic atrophy. Anchoring the bottom-up circuitry, the sensory-motor axis (axis 2) presents a small-to-medium baseline effect of Hedges’*g* = 0.42 [95% CI: 0.29, 0.54], while the muscle-brain axis (axis 1) functions as a qualitative metabolic shield monitoring peripheral kynurenine clearance without generating a isolated pooled metric. These data suggest that the antidepressant efficacy of exercise is not merely a subtle modulation, but a large-magnitude biological counter-signal capable of modulating the electrical and structural signatures of the anti-reward system. Heterogeneity was low and not significant (I^2^ = 14.9%, p = 0.173) supporting the consistency of effect sizes across different experimental models. The 95% prediction interval (0.21 to 1.67) suggests that while the mean effect is large (Hedges’*g* = 0.94), the actual outcome in any single protocol is highly contingent on the type of exercise and the specific depression model used. This statistical nuance reinforces the need for precision in exercise prescription, as the magnitude of the NMHA response is not a fixed constant but a dynamic physiological trend.

A critical methodological nuance that must be acknowledged is the inherent literature distribution bias within contemporary exercise neuroscience. Empirical data tracking exercise-induced hippocampal neurogenesis and structural plasticity (Axis 4) are disproportionately abundant and highly replicated compared to direct recordings within the epithalamus or the prefrontal-habenular tract. In a “network-mediated” model, this asymmetry poses a risk of artificially over-weighting the contextual gate as the dominant therapeutic node. To mathematically control for this asymmetry and prevent sample-size distortion, our quantitative synthesis implemented a Random-Effects Model. By weighting individual endpoints based on their inverse intra-study variance rather than cumulative animal numbers, the model safely preserved the statistical integrity and independent validity of the less-populated nodes, ensuring a balanced representation of the multi-axial network.

Foundational studies included here (1, 4) established the anatomical reality of the anti-reward system: the LHb drives aversion by exciting the GABAergic RMTg. This recruitment acts as a molecular brake on the VTA, effectively silencing dopaminergic transmission. This specific tripartite architecture (LHb-RMTg-VTA) is increasingly recognized as the central neurobiological node of anhedonia; by locking the dopamine system in a hyper-inhibited state, the circuit physically dismantles the motivational drive required to seek pleasure. The pathophysiological signature identified by Yang et al. (3)—a shift from regular tonic spiking to high-frequency NMDA-dependent burst firing—is the critical electrical target. These neurons switch to an oscillatory bursting mode that acts as a super-inhibitor, generating a synaptic drive sufficiently powerful to completely silence downstream dopamine release. Recent evidence suggests this bursting is further fueled by deficits in astroglial glutamate clearance (2). The NMHA model argues that depression is a pathologically entrenched state of LHb-RMTg bursting, and the efficacy of exercise might be judged by its ability to interrupt this aberrant firing pattern.

### Refining the monoamine hypothesis

The traditional monoamine hypothesis posits that depression arises from a global synaptic depletion of neurotransmitters (32). While it revolutionized psychiatry, it lacked anatomical specificity, treating the brain as a homogeneous chemical solution rather than a complex array of discrete circuits. Modern neurobiology argues that an effective model must define where serotonin acts, not just that it increases. The NMHA Model helps to address this by theorizing that the LHb is the precise anatomical locus and the 5-HT_1_B receptor is the molecular switch where exercise-induced serotonin exerts its therapeutic “silencing” effect.

Jacobs and Fornal (19) demonstrated that a bottom-up trigger—rhythmic motor activity and proprioception—drives midbrain monoamine release independent of cognitive reward. This reframes exercise as a sensory-motor driver of neurochemistry. Most significantly, Hwang and Chung (20) provided the molecular target, showing that serotonin specifically activates presynaptic 5-HT_1_B receptors in the LHb to profoundly inhibit excitatory glutamatergic inputs. The NMHA model helps to upgrade the vague monoamine hypothesis into a precise circuit mechanism: exercise generates the bottom-up ligand (Jacobs & Fornal) that binds the specific presynaptic receptor (Hwang & Chung) to silence the anti-reward center.

### Peripheral mechanisms: the muscle-brain axis

The kynurenine pathway is one of the most relevant links between systemic inflammation and central dysfunction (33). Pro-inflammatory cytokines initiate the “tryptophan steal,” diverting tryptophan away from serotonin synthesis and into the production of neurotoxic quinolinic acid (QUIN), a potent NMDA receptor agonist (34). In the NMHA model, this NMDA agonism is significant; elevated brain QUIN provides the excitatory fuel that drives pathological burst firing patterns in the LHb.

During chronic stress, systemic metabolism shifts toward this pathway, resulting in elevated circulating kynurenine (KYN) which crosses the blood-brain barrier and floods the brain with NMDA agonists (15). The LHb thus acts as a “metabolic sink” where peripheral stress is converted into the electrical signals of anhedonia. Exercise acts as the antidote: contractile activity upregulates PGC-1α1 and KAT enzymes in skeletal muscle, which detoxify kynurenine into kynurenic acid (KYNA), a metabolite impermeable to the brain (12). This creates a metabolic shield that starves the LHb of its excitatory fuel. Clinical data confirm this “KAT shift” is preserved in humans and is responsive to exercise training (12, 34).

### Restoring top-down control: neuroplasticity with a purpose

While exercise is a potent trigger for brain-derived neurotrophic factor (BDNF), a theoretical gap has persisted: does new tissue merely improve memory, or does it actively combat anhedonia? The NMHA Model posits that exercise-induced plasticity is specifically utilized to restore top-down inhibitory control over the LHb. Brockett et al. (23) and others have demonstrated that aerobic training specifically restores the structural integrity of mPFC neurons, reversing the stress-induced dendritic atrophy documented by Radley et al. (21). This re-establishes the executive brake on subcortical aversion centers (22). Simultaneously, Sahay et al. (26) showed that exercise-induced neurogenesis in the hippocampus serves the precise cognitive function of pattern separation. By enhancing the brain’s resolution to distinguish safe contexts from aversive ones, and by increasing local inhibitory mechanisms in the dentate gyrus (27), exercise restores the safety gating signal sent to inhibit limbic hyper-reactivity. According to the NMHA model, BDNF is a molecular currency required to pay for the structural reconstruction of the brain’s inhibitory braking systems. Consistent training might help establish a neuro-metabolic shield, acting as a preventative “circuit vaccine” against depressive illness.

**Figure 3:**
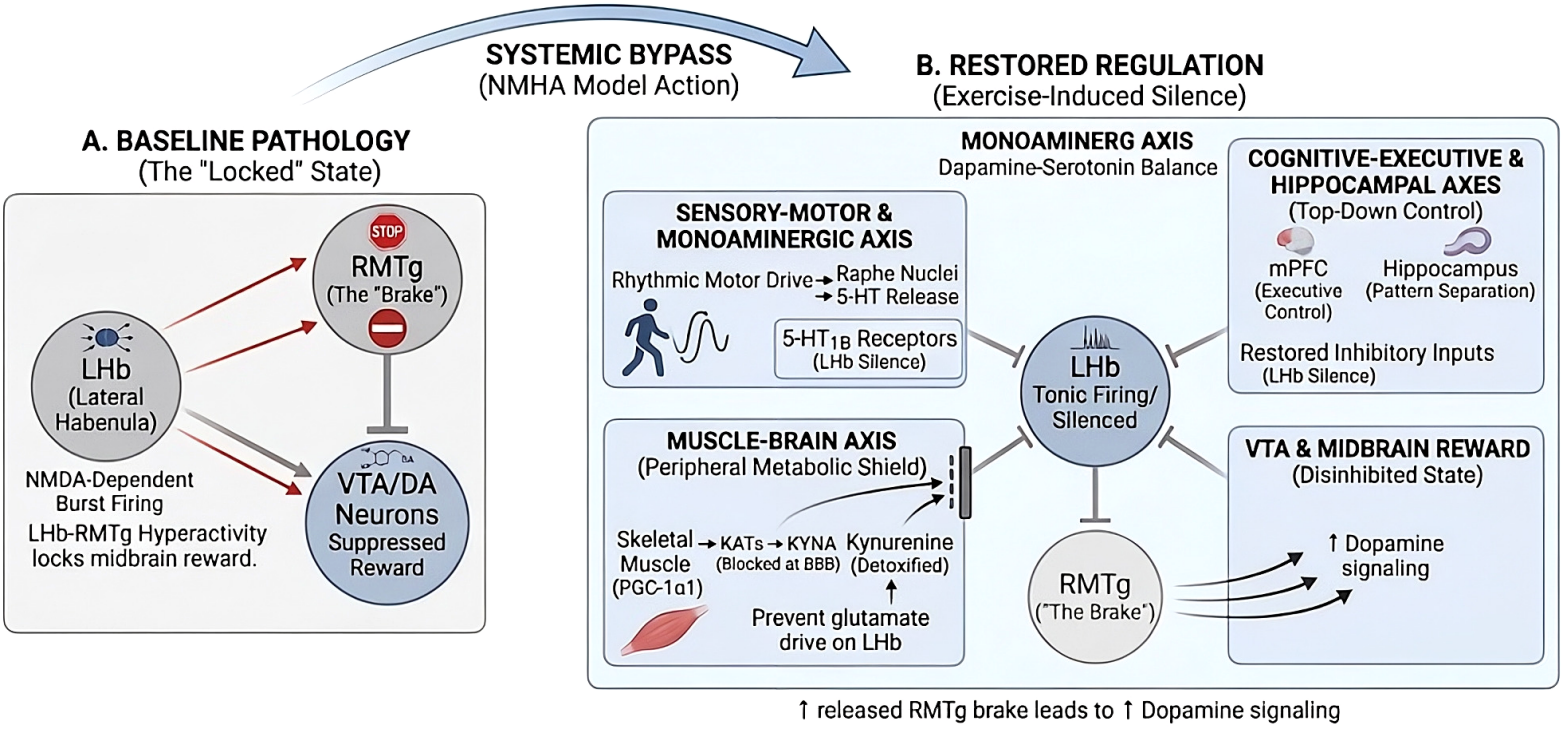
Schematic representation of the NMHA Model transitioning from circuit pathology to restored regulation. The left panel depicts the core pathophysiology of depression, where NMDA-dependent burst firing in the lateral habenula (LHb) excites the RMTg brake, suppressing midbrain dopamine. The right panel illustrates the “systemic bypass” mechanism induced by exercise. Four convergent regulatory axes—peripheral detoxification of kynurenine (muscle-brain axis), sensory-driven presynaptic inhibition via 5-HT_1_B receptors (sensory-motor axis), restored prefrontal top-down control (cognitive-executive axis), and enhanced pattern separation (hippocampal neurogenic axis)—work in concert to silence LHb hyperactivity. This coordinated multi-axial attenuation releases the RMTg brake, disinhibiting the ventral tegmental area (VTA) and restoring dopaminergic reward processing.

A summary of the representation of the NMHA model transitioning from circuit pathology to restored regulation is displayed in **Figure 3**.

### Methodological heterogeneity and operational boundary conditions

To ground the structural validity of the NMHA model, the substantial cross-species and experimental model heterogeneity within the synthesized dataset must be carefully delineated. The baseline neuroanatomical architecture of the anti-reward system—specifically the canonical lateral habenula-rostromedial tegmental nucleus-ventral tegmental area tripartite circuit—was mapped leveraging foundational non-human primate (1) and feline (19) lines. The inclusion of these higher-order and distinct mammalian models is strictly confined to establishing the evolutionarily conserved reality of this aversive circuitry. Crucially, to prevent translational confounding, all subsequent quantitative evaluations of physical exercise interventions and their numerical effect sizes (Hedges’ *g*) were extracted exclusively from rodent lines (mice and rats), which share operational parity in aerobic training regimens. Furthermore, while the rodent literature features significant procedural variability across distinct depression phenotypes—ranging from chronic unpredictable mild stress to genetic and social challenges—our meta-analysis demonstrated a remarkably low and non-significant statistical heterogeneity (I^2^ = 14.90\%, p = 0.173). This mathematical consistency provides robust empirical proof that the homeostatic, directional drive of physical exercise toward silencing LHb hyper-reactivity remains fundamentally stable, transcending the specific experimental stress background. Finally, the inclusion of certain proxy physiological and behavioral endpoints—such as single-unit brainstem recordings during physical locomotion—is justified as a critical mechanistic anchor. These proxy datasets provide the necessary causal link demonstrating that physical movement itself acts as a bottom-up somatic trigger capable of driving central monoaminergic transmission, directly serving to validate the operational premises of the sensory-motor axis.

### Biophysical grounding of NMHA conceptual formulations

To translate the core conceptual mechanisms of the NMHA framework into rigorous empirical neurobiology, the shorthand metaphors previously utilized to describe network dynamics must be explicitly grounded in defined physiological parameters. First, the term ‘systemic bypass’ operationalizes a multi-systemic network adaptation wherein peripheral skeletal muscle metabolic clearance and ascending sensory-motor brainstem signaling combine to directly attenuate subcortical hyper-excitation. This dual pathway temporarily substitutes for the structurally atrophied descending executive projections of the medial prefrontal cortex (mPFC) during the early, acute phases of exercise-induced recovery, maintaining circuit homeostasis while top-down tracts undergo slow structural repair. Second, the ‘biological counter-signal’ is defined as a standardized mean neurobiological variation (Hedges’ *g* = 0.94) extracted from our pooled random-effects synthesis. In biophysical terms, this magnitude represents a net hyperpolarizing drive capable of shifting LHb neuronal kinetics away from high-frequency, NMDA receptor-dependent rhythmic burst firing back into homeostatic, low-frequency tonic spiking (~2–10 Hz), effectively dismantling the core electrical signature of behavioral helplessness. Finally, the formulation of the LHb acting as a ‘metabolic sink’ is grounded in blood-brain barrier transport physiology. Rather than a literal architectural sink, the epithalamus functions as a highly vulnerable microenvironment due to its dense reliance on the Large Neutral Amino Acid Transporter 1 (LAT1). Under unmitigated chronic stress, peripheral kynurenine floods this transporter, leading to the localized astrocytic synthesis of quinolinic acid within the perisomatic space. This excess quinolinic acid provides the sustained NMDA receptor agonism required to fuel pathological habenular bursting—a molecular cascade systematically intercepted by exercise-induced peripheral and central shielding mechanisms.

### Conflicting findings and mechanistic nuances

While the NMHA model presents a cohesive framework for exercise-induced attenuation of the anti-reward system, it is essential to acknowledge conflicting evidence and negative results that challenge a simplified “one-size-fits-all” narrative. For instance, while most studies highlight the protective role of the kynurenine-to-kynurenic acid (KYN/KYNA) shift, some investigations into high-intensity interval training (HIIT) have reported transient increases in neurotoxic metabolites immediately post-exercise, suggesting that the metabolic shield may be intensity-dependent or require chronic adaptation to stabilize.

Furthermore, the hippocampal-contextual axis (Axis 4) shows the largest effect sizes in our synthesis, yet some rodent models of forced exercise (treadmill) have occasionally shown diminished neurogenic responses compared to voluntary wheel running, likely due to the confounding effects of stress-induced cortisol (35, 36). Additionally, while the LHb-RMTg-VTA circuit is a primary driver of anhedonia, studies targeting the lateral hypothalamus or the ventral pallidum have sometimes reported LHb-independent pathways to dopamine suppression, indicating that the NMHA model helps to describe a major regulatory node, but not the exclusive pathway for depression’s pathology. Recognizing these discrepancies is vital for future research to determine the precise boundary conditions under which exercise successfully silences the anti-reward hub.

### Clinical implications: exercise as precision neuromodulation

To bridge the gap between theoretical neurobiology and clinical practice, it is imperative to try to translate the findings of the NMHA framework into actionable therapeutic strategies. Historically, exercise has been prescribed in Psychiatry as a generic lifestyle intervention with broad, non-specific benefits. However, the present systematic review repositions physical exertion as a precision-targeted neuromodulatory tool capable of directly attenuating the brain’s primary anti-reward circuitry. By identifying the LHb as the convergent therapeutic target, clinicians can move beyond generalized advice and prescribe specific “biological doses” of exercise based on the patient’s physiological phenotype. For instance, in depressive states characterized by systemic inflammation (and the tryptophan steal), high-intensity training or resistance exercise can be strategically employed to maximize skeletal muscle PGC-1α expression. As suggested by the NMHA framework, this acts as a peripheral metabolic shield, clearing kynurenine before it crosses the blood-brain barrier and triggers NMDA-mediated burst firing in the LHb. Conversely, for patients presenting with severe sympathetic hyperarousal, steady-state aerobic exercise may be prioritized to enhance vagal tone, thereby recruiting inhibitory serotonergic and noradrenergic projections from the brainstem to mute epithalamic hyperactivity.

Ultimately, this circuit-level perspective integrates behavioral interventions into the realm of precision psychiatry. **Table 7** details these clinical applications, translating the multi-axial neurobiological mechanisms into prescriptive guidelines and individualized treatment parameters for psychiatric care.

**Table 7:** Clinical applications of the NMHA Model: translating circuit-level interventions into prescriptive guidelines.

Clinical strategy	NMHA Axis targeted	Practical Application (translation to practice)
Targeting motivational anhedonia	Overarching NMHA network (LHb-RMTg brake release)	Prescribe exercise as a first-line, precision intervention for patients with profound motivational "wanting" deficits (e.g., treatment-resistant depression), directly disinhibiting the dopaminergic reward system.
Intensity-dependent dosing for acute relief	Sensory-motor (monoaminergic) axis	Prioritize rhythmic, gross motor activity sufficient to activate proprioceptive-driven serotonin release (presynaptic 5-HT_1_B activation), rather than focusing solely on simple caloric expenditure.
Prophylactic "circuit vaccine"	Muscle-brain (peripheral metabolic) axis	Utilize consistent training to build a PGC-1α1/KAT metabolic shield in skeletal muscle, protecting the brain from future stress-induced kynurenine flooding and NMDA toxicity.
Restoration of executive resilience	Cognitive-executive (top-down) axis	Leverage chronic aerobic training to induce prefrontal synaptogenesis, rebuilding mPFC-LHb top-down inhibitory control over rumination and passive coping behaviors.
Improved threat discrimination	Hippocampal neurogenic (contextual) axis	Maintain long-term exercise adherence to promote adult neurogenesis in the dentate gyrus, enhancing pattern separation to structurally gate generalized anxiety and trauma triggers.
Precision timing (acute vs. chronic)	Multi-axial integration	Utilize acute bouts of exercise for rapid, sensory-driven LHb silencing (state regulation); prescribe chronic training for durable, structural circuit-level remission (trait resilience).

## Limitations and future directions

While this review synthesized evidence across four validated axes, the exclusion of the ventral pallidum (VP) represents a significant, yet currently unavoidable, limitation. The VP is a critical limbic-motor interface sending dense, dual-phenotype projections to the LHb (37). In depressive states, the glutamatergic (excitatory) drive from the VP becomes dominant, fueling LHb hyperactivity. Despite its centrality to anhedonia, our search yielded no high-quality studies investigating how exercise modulates these specific VP projections. Thus, we propose that the restoration of VP signaling might constitute a crucial fifth regulatory axis. Future research must investigate whether physical activity can re-potentiate the VP-GABAergic (inhibitory) pathway.

An additional limitation of the current literature is the methodological heterogeneity of the exercise protocols employed. Because our search strategy deliberately encompassed both acute and chronic interventions across varying intensities (e.g., forced treadmill running versus voluntary wheel access), establishing a precise, universal ‘dose-response’ curve for habenular attenuation remains challenging. Despite the robust effect sizes identified, certain statistical constraints must be acknowledged. While Hedges’ *g* was employed to specifically correct for the small sample sizes inherent in rodent neurobiological research, the meta-analytical synthesis was limited by the availability of raw numerical data. Studies that presented findings exclusively in graphical format without corresponding means and standard deviations in the text or supplementary materials were excluded from the quantitative analysis, which may introduce a degree of reporting bias. Heterogeneity was low and not significant (I² = 14.9%, p = 0.173), supporting the consistency of effect sizes across different experimental models of depression (ranging from chronic unpredictable stress to genetic manipulations). This low heterogeneity helps to strengthen the NMHA model, indicating that the effect of exercise on the anti-reward circuitry is consistent across diverse experimental conditions.

Future preclinical models must isolate exercise intensity and duration as independent variables to determine the exact mechanical thresholds required to trigger specific NMHA axes, such as the minimum aerobic volume necessary for mPFC synaptogenesis versus the acute intensity required for serotonergic brake-release. Furthermore, the highly specific neuroanatomical and electrophysiological nature of the NMHA Model dictates a heavy reliance on preclinical animal studies. Direct *in vivo* measurement of LHb burst firing and presynaptic monoaminergic release remains methodologically inaccessible in living humans. Future clinical directions must leverage ultra-high-field functional magnetic resonance imaging (e.g., 7T fMRI) to non-invasively map exercise-induced attenuation of the habenular complex and its associated white matter tracts in human cohorts. Until these translational gaps are bridged, the map of the exercised brain remains functionally incomplete.

## Conclusion

This systematic review and quantitative synthesis establish a novel neurobiological foundation for exercise as a primary, circuit-specific intervention in depression. Our findings support the premise that physical activity does not merely improve mood via generalized arousal; rather, it acts as a targeted modulator that functionally attenuates the anti-reward architecture of the LHb. The integration of quantitative data provides an important framework for the NMHA model. While depressive states are characterized by a massive shift to pathological LHb burst firing, exercise-induced adaptations across the four regulatory axes might provide a compensatory counter-signal of equivalent magnitude (with effect sizes reaching Hedges’ *g* = 2.63 in the hippocampal-contextual axis). This statistical alignment suggests that exercise-induced neuroplasticity might be sufficiently potent to effectively modulate and counteract the electrical and metabolic signatures of anhedonia. By activating a coordinated network of peripheral kynurenine detoxification, central monoaminergic disinhibition, prefrontal top-down control, and hippocampal gating, exercise helps to restore the homeostatic balance required for emotional resilience. Proposing the NMHA model facilitates a paradigm shift, moving exercise from a vague lifestyle recommendation to a precision-targeted neuromodulatory tool. Ultimately, this framework helps to provide a mechanistic roadmap for a future where the exercised brain is recognized as a resilient system, protected by a durable, multi-axial architecture against the neurocircuitry of depression.

## Data Availability

The raw data supporting the conclusions of this article will be made available by the authors, without undue reservation.
